# Mood disorders and parity – A clue to the aetiology of the postpartum trigger

**DOI:** 10.1016/j.jad.2013.09.034

**Published:** 2014-01

**Authors:** Arianna Di Florio, Lisa Jones, Liz Forty, Katherine Gordon-Smith, Emma Robertson Blackmore, Jess Heron, Nick Craddock, Ian Jones

**Affiliations:** a Institute of Psychological Medicine and Clinical Neurosciences, MRC Centre for Neuropsychiatric Genetics and Genomics, Cardiff University School of Medicine, Hadyn Ellis Building, Maindy Road, Cathays, Cardiff CF24 4HQ, UK; b Department of Psychiatry, School of Clinical and Experimantal Medicine, University of Birmingham, UK; c University of Rochester Medical Center, School of Medicine and Dentistry, USA

**Keywords:** Women mental health, Postpartum mood disorders, Bipolar disorder, Recurrent major depression

## Abstract

**Background:**

Episodes of postpartum psychosis have been associated with first pregnancies in women with bipolar I disorder. It is unclear, however, if the effect extends to episodes at other times in relation to childbirth and to women with other mood disorders such as major depression and bipolar II disorder. This primiparity effect, which is also seen in other pregnancy related conditions such as pre-eclampsia, is a potentially important clue to the aetiology of childbirth related mood episodes.

**Methods:**

Participants were interviewed and case notes reviewed. Best-estimate diagnoses were made according to DSM-IV criteria. Data on the occurrence of episodes in pregnancy and the postpartum were available on 3345 full term deliveries from 1667 participants, 934 with bipolar I disorder (BD-I), 278 with bipolar II disorder (BD-II) and 455 with recurrent major depression (RMD).

**Results:**

Onsets of psychosis/mania within 6 weeks of childbirth were overrepresented in primiparae (*p*=0.007) with BD-I. Although primiparity was not associated with perinatal bipolar depression, there was an association with the onset of depression within 6 weeks in women with RMD (*p*=0.035). Whilst women experiencing a postpartum episode were less likely to go on to have further children, this did not account for the association with primiparity.

**Limitations:**

Data were collected retrospectively. Information on pharmacological treatment was not available.

**Conclusions:**

Primiparity is associated not only with postpartum psychosis/mania in BD-I, but also with postpartum depression in RMD. Psychosocial factors and biological differences between first and subsequent pregnancies may play a role and are candidates for examination in further studies.

## 1 Background

The link between childbirth and severe mood disorders has been recognized for hundreds of years. Episodes in pregnancy and the postpartum affect 2 in 3 women with mood disorders and have serious consequences not only for the woman, but also for her baby and entire family (Di Florio et al., 2013).

Understanding the triggering of severe postpartum mood episodes is therefore of vital importance. An intriguing clue that may help in this task is the greater risk following first pregnancies that has been shown in a number of previous studies (Blackmore et al., 2006; Kendell et al., 1981; Kirpinar et al., 1999; Thomas and Gordon, 1959; Videbech and Gouliaev, 1995). This has implications, not only for the identification of women at risk, but also for the aetiology of mood disorders occurring at this time. The link to primiparity may suggest relationships with other pregnancy related disorders such as pre-eclampsia in which parity is known to play an important role and lead to specific hypotheses about the nature of the postpartum trigger.

In both register based (Kendell et al., 1981, Videbech and Gouliaev, 1995) and clinical studies (Bergink et al., 2011a, 2011b; Blackmore et al., 2006; Kirpinar et al., 1999; Thomas and Gordon, 1959) postpartum psychosis has been shown to be more common after first deliveries. The link between first pregnancy and non-psychotic postpartum depression is more controversial. The majority of studies report no effect of parity (Kendell et al., 1981; Marks et al., 1991), although one study did find an association with primiparity (Glavin et al., 2009) and another found the opposite effect (Milgrom et al., 2008).

A number of methodological issues may influence the results of studies on parity. If women suffering a postpartum episode are less likely to go on to have further children, this will reduce rates in multiparae and may account for the association with first pregnancies. Similarly, other demographic and obstetric variables may also explain the excess of psychosis in primiparae. For example, first pregnancies occur at a younger age and delivery by caesarean section has been associated with both primiparity (Roman et al., 2008) and postpartum psychosis (Kendell et al., 1981), although the findings for postpartum psychosis are controversial (Valdimarsdóttir et al., 2009). Another issue is the variability in methodology in studies of postpartum mood disorder. In particular this may account for the heterogeneous results with perinatal depression and parity. There is a lack of consensus on the definition of postpartum depression and it has been argued that parity itself influences the validity of rating scales for perinatal depression (Ji et al., 2011).

Despite the potential importance of this topic, there is a paucity of large-scale systematic studies on the effect of parity in women with severe mood disorders and with few exceptions (Blackmore et al., 2006), the research to date has not considered potential confounders. Moreover, although women with bipolar disorder experience both postnatal depression and postpartum psychosis, no data are available on parity and bipolar depression.

In this paper we test the hypothesis that the risk of perinatal mood episodes is greater following first pregnancies and ask whether this association: (i) holds across the mood disorder spectrum (bipolar I – BD-I, bipolar II – BD-II and recurrent major depression – RMD); (ii) is found for both episodes of high and low mood; (iii) applies to all episodes in relation to pregnancy and childbirth or is limited to those with onset in the immediate postpartum; and finally (iv) is impacted by possible confounders, such as decisions about having further children, age at pregnancy and method of delivery.

## 2 Methods

### 2.1 Sample

Participants were drawn from 2 clinical and genetic studies of mood disorders (one on RMD and one on BD) and are described in detail elsewhere (Cohen-Woods et al., 2009; Raybould et al., 2005). In brief, participants were recruited using both systematic and non-systematic methods across the United Kingdom. Systematic recruitment involved the screening of records of Community Mental Health Teams, in order to identify patients with major affective disorders with all patients deemed suitable for inclusion invited to participate. Non-systematic recruitment methods included advertisements in general practitioner surgeries, the media and via patient support organizations (Depression Alliance and Bipolar UK). In the current analyses, 26% of the sample was recruited systematically. No differences emerged in demographic variables between systematically and not systematically recruited women.

All participants were aged 18 years or over. Patients were excluded from the original studies if they: (i) had a lifetime diagnosis of intravenous drug dependency; (ii) had only experienced affective illness as a result of alcohol or substance dependence; and (iii) had only experienced affective illness secondary to medical illness or medication.

The major depression study aimed to recruit a sample of participants with clear-cut unipolar depression. Thus, individuals in the RMD group were excluded if they (i) had a first or second degree relative with a clear diagnosis of bipolar affective disorder or schizophrenia, schizotypal disorder, persistent delusional disorder, acute and transient psychotic disorders or schizoaffective disorder, or (ii) had ever experienced mood incongruent psychosis or psychosis outside of mood episodes.

Participants were included in the current analyses if they (i) had a lifetime diagnosis of DSM-IV BD-I or BD-II or RMD (ii) had at least one full term delivery. As we were interested in mood disorder episodes in the reproductive years, women were excluded from the current analyses if they reported an age of onset in the post-menopausal period. A cut-off of 50 years old was set, according to the mean European age at menopause.

This study received all necessary Multi-Region and Local Research Ethics Committee approvals. After complete description of the study to the subjects, written informed consent was obtained.

### 2.2 Procedures and diagnostic criteria

Participants were interviewed using the Schedules for Clinical Assessment in Neuropsychiatry (SCAN) (Wing et al., 1990), which provides detailed information about lifetime psychopathology. Psychiatric and general practice case-notes where available were also reviewed. Based on these data best-estimate lifetime diagnoses were made according to DSM-IV criteria (American Psychiatric Association, 2000) and key clinical variables, such as age at onset and number of episodes, were rated. In cases where there was doubt, diagnostic and clinical ratings were made by at least two members of the research team blind to each other's rating. Inter-rater reliability was formally assessed using 20 cases. Mean kappa statistics were 0.85 for DSM-IV diagnoses and ranged between 0.81 and 0.99 for other key clinical categorical variables; mean intra-class correlation coefficients were between 0.91 and 0.97 for key clinical continuous variables. Team members involved in the interview, rating and diagnostic procedures were all research psychologists or psychiatrists.

Pregnancy and postpartum mood episodes were defined for each delivery according to the symptomatology and the time of onset. Depression was defined as an episode of DSM-IV non-psychotic major depression, while mania/psychosis was defined as an episode of DSM-IV mania or DSM-IV psychotic depression. In the current diagnostic systems for psychiatric disorders (DSM-IV and ICD-10 (World Health Organization, 1992)), postpartum mood episodes are not separate nosological entities. The DSM-IV has an onset specifier that includes all mood episodes with an onset within 4 weeks after childbirth, while ICD-10 has a postpartum category for episodes with onset within 6 weeks, but this diagnosis should only be used for episodes that do not meet the criteria for disorders that can be classified elsewhere. Current diagnostic systems do not allow episodes in pregnancy or later in the postpartum to be linked to childbirth. However, rather than the 4 or 6 weeks cut-off, a 6 month or 1 year postpartum period is commonly used in clinical practice. Moreover, we have shown that the familiality for postpartum depression maximizes with a 6–8-week criterion of postpartum onset and thus the DSM-IV onset specifier may be to narrow (American Psychiatric Association and DSM-IV, 2000). In this study we grouped the episodes with onsets: (a) in pregnancy; (b) in the first 6 weeks postpartum; and (c) between 6 weeks and 6 months after childbirth.

Several obstetric variables have been related to postpartum mood disorders, especially mania/psychosis (Blackmore et al., 2006), and therefore information on the sex of the baby, age at pregnancy and method of delivery was collected.

### 2.3 Statistical analyses

Statistical analyses were performed using R version 2.13.0 (Copyright 2011 by The R Foundation for Statistical Computing).

Deliveries from the same woman may not be considered as independent events. So we applied the univariate McNemar's test to 2×2 contingency table and multivariate mixed logistic regression to test the hypothesis that the proportion of first perinatal periods affected by mood episodes is greater than that of second perinatal periods.

Other independent univariate comparisons were performed using contingency tables and Chi-Squared statistics or Fisher Exact test for categorical variables, *t*-test for normally distributed quantitative variables and non-parametric rank tests for those not normally distributed.

Information on time of onset of perinatal episodes was available for 90% of deliveries affected. The software Amelia II (Honaker et al., 2011) was used to perform multiple imputations. This approach has been reported to reduce bias and to be more efficient than list-wise deletion (Honaker et al., 2011). The data sets obtained were then analysed using the package Zelig (http://GKing.harvard.edu/zelig).

## 3 Results

### 3.1 Sample characteristics

Detailed information was collected for 3345 full term deliveries from 1667 participants, 934 with BD-I, 278 with BD-II and 455 with RMD.

Sample characteristics are summarized in Table 1. We have previously reported the occurrence of perinatal episodes in parous women recruited in our studies (Di Florio et al., 2013). The morbidity rates reported here exceed to some extent the figures of our previous study, which focused on the prevalence of perinatal mood episodes and therefore excluded women recruited on the basis of a history of postnatal psychosis or depression. In the present study we included such women, and found that 1065/3345 (31.8%) deliveries across all women in out sample were complicated by non-psychotic depression during pregnancy or after childbirth and 524/3345 (15.7%) by mania/psychotic depression.

### 3.2 Parity

Data were available for 1667 first and 1678 subsequent pregnancies. In the BD-I group, 327 (35.0%) women reported an episode of mania/psychotic depression in the first pregnancy and postpartum period. The proportion dropped to 20.5% (111/541) in relationship to the second pregnancy and to 14.6% (30/205) and 14.8% (12/81) in relation to subsequent pregnancies. Women with BD-I reported similar rates of depression across all pregnancy and postpartum periods (range 21.0–24.2%).

Rates of depression in women with BD-II dropped between the first (46.0%, 128/278) and the second pregnancy (33.0%, 63/191). Large confidence intervals allowed less precise estimates for subsequent pregnancies.

An episode of depression occurred in 48.1% (219/455) primiparae with RMD. The proportion dropped to 37.3% (125/335) in the second pregnancy and postpartum period and to 33.5% (55/164) and 27.4% (17/62) in relation to subsequent pregnancies. We do not report here the estimates on pregnancies affected by depressive psychosis in the BD-II group and in the RMD group because of the low incidence rates (Table 1).

### 3.3 Controlling for the influence of perinatal episodes on having further children

The psychiatric outcome of first pregnancies was significantly associated with the proportion of women going on to have further children. In the BD-I group, only 54.0% of primiparae experiencing a childbirth related mania/depressive psychosis had further children compared to 73.2% of those who did not have a first pregnancy affected (OR: 2.3, 95% CI 1.70–3.16, *p*<0.001). Similarly primiparae with either BD-II or RMD who suffered episodes of childbirth related non-psychotic depression were less likely to have further children (respectively 72.2% vs. 86.0%, OR: 2.4, 95% CI 1.22–4.68, *p*=0.008 and 76.8% vs. 86.2%, OR: 1.9, 95% CI 1.11–3.20, *p*=0.013).

It is therefore possible that any association between mood episodes and primiparity is due to women who suffer severe postpartum episodes deciding not to have further children. To examine this potential bias, we compared the rates of mood episodes in the first perinatal period with the rates of mood episodes in the second perinatal period in a subsample of women who were (a) multiparous and (b) had experienced at least one delivery affected by a mood episode. There were 185 multiparae with BD-I and at least one episode of perinatal mania or psychotic depression. We focused our analysis on episodes occurring with onset within 6 weeks postpartum, as the vast majority (90%) of these episodes occurred within this timeframe. Primiparity was significantly associated with mania/psychotic depression occurring within 6 weeks of delivery (*p*=0.001, OR 2.0, 95% CI 1.64–2.56).

No association between parity and perinatal depression was found in the group of 93 multiparae with BD-II and at least one pregnancy affected by perinatal depression (*p*=0.137 for depression in pregnancy, *p*=0.473 for depression within 6 weeks postpartum, *p*=0.096 for depression occurring between 6 weeks and 6 months postpartum).

**Fig. 1:**
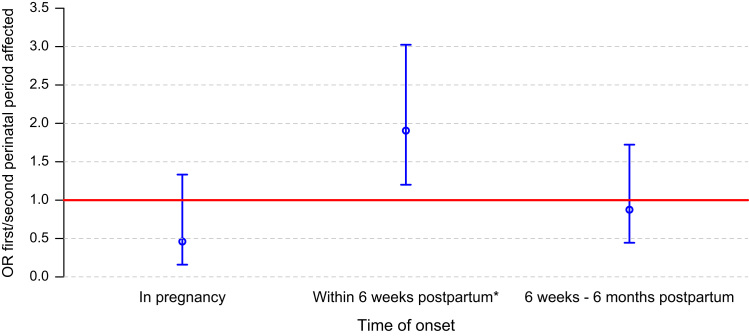
Association between parity and perinatal depression by time of onset in women with RMD. Only multiparae with at least one pregnancy affected were included (*N*=193). Depression was defined as an episode of DSM-IV non-psychotic major depression. Odds ratios were used to quantify the association between parity and perinatal affective episodes. If odds ratios are greater than one then perinatal depression is more likely to happen in the first perinatal period than in the second perinatal period. *Significant effect (*p*=0.003).

In the RMD group (*N*=193), primiparity was associated with depression with onset within 6 weeks of childbirth (*p*=0.003, OR 1.8, 95% CI 1.49–2.25), but not with onset in depression in pregnancy (*p*=0.653) or later in the postpartum (*p*=0.447; Fig. 1).

### 3.4 Controlling for the influence of age and Caesarean section

The association between primiparity and severe mood episodes could be mediated by age at pregnancy. If this were true, we would expect women to report a younger age at delivery for pregnancies complicated by psychiatric sequelae. However, there were no age differences between deliveries complicated by postnatal psychosis (mean age at delivery 26.0, sd 4.99), those complicated by postnatal depression (mean age at delivery 26.0, sd 4.94) and those with no psychiatric complications (mean age at delivery 26.0, sd 5.02).

In some previous studies (e.g. Kendell et al., 1981), deliveries by Caesarean section were associated with postpartum psychosis. In our sample method of delivery was not associated with postpartum mania/psychosis in BD-I (*p*=0.626) or with postpartum non-psychotic depression (*p*=0.443) therefore an association with Caesarean section does not account for the affect of parity we have observed.

## 4 Discussion

In this study we explored the link between parity and mood episodes. The results support previous research findings of an association between primiparity and mania/psychosis occurring soon after childbirth (e.g. Kendell et al., 1981). In women with BD-I, we found an excess of postpartum mania/psychosis following first deliveries, but no similar link to primiparity for episodes of non-psychotic depression. In contrast we found that there was a greater risk of a postnatal depression following first deliveries in women with RMD. We did not find an effect of parity in women with a lifetime diagnosis of BD-II.

The association between parity and mood episodes was significant only for episodes occurring within 6 weeks postpartum. Episodes occurring in pregnancy or later in the postpartum did not show a significant association with parity (Table 2).

The excess of mood episodes in primiparae was not explained by women with a postpartum episode being less likely to go on to have further children, by the age at pregnancy or by method of delivery.

### 4.1 What accounts for the influence of parity?

There are both psychosocial and biological differences between first and subsequent pregnancies that might underpin the association with parity. Women having their first child experience higher levels of stress and the concerns of motherhood are different for first and subsequent deliveries (Hung, 2007). However, while undoubtedly important for postpartum mood disorders in general, in the case of severe postpartum episodes, there is a lack of evidence implicating psychosocial factors (Brockington et al., 1990; Marks et al., 1991, Dowlatshahi and Paykel, 1990).

Another possible factor that could lead to the primiparity effect we have observed is prophylactic strategies having an influence following further pregnancies. Pregnant women with established mood disorder may be aware of the risk of recurrence and take medication to prevent or promptly treat an emerging postpartum episode. A reduced risk in multiparae may therefore be due to the effects of prophylactic medication and other strategies designed to help keep women well that are known to be at high risk.

Biological differences, immunological or hormonal for example, between first and subsequent pregnancies are also important candidates for future studies that may, at least in part, explain the association between parity and severe mood episodes.

### 4.2 Implications for further research

An association with parity has been reported for other pregnancy/delivery related disorders. Pre-eclampsia has robustly been associated with primiparity (Robillard et al., 2011), while more controversial findings have reported that autoimmune disorders (Bülow Pedersen et al., 2006; Cockrill et al., 2010; Greer et al., 2011) and gestational diabetes (Dode et al., 2009) are overrepresented in multiparous women.

There are a number of important overlaps in the clinical presentation and epidemiology of pre-eclampsia and postpartum psychosis and this relationship has been recognized for over 160 years (Reid, 1848). Associations between pre-eclampsia and mood episodes have been described (Cripe et al., 2011), and psychosis can be a dramatic feature of eclampsia (Brockington, 2006). It is of interest that psychotic symptoms occurring in this condition are not merely a post-ictal phenomenon but rather occur prior to this end stage and are therefore thought to be part of the systemic effects of pre-eclampsia on the central nervous system (Brockington, 2006).

Our findings give clues to the pathogenesis of the postpartum trigger and, perhaps, to the causation of mood disorders more generally. Future studies are needed to clarify the role that biological and psychosocial differences between first and subsequent pregnancies may have on the postpartum triggering of mood episodes. Studies exploring the relationship of perinatal mood disorders and other conditions associated with parity such as pre-eclampsia may also prove fruitful.

Clinical studies exploring the effect of parity on mood disorders should take into account the possible effect of medication in reducing the risk of a perinatal relapse.

### 4.3 Implications for nosology

In this study we refined the definition of childbirth related episodes to better establish the relationship between parity and mood disorders. In women with both BD-I and RMD, the relationship with parity was only significant for episodes with onset within 6 weeks of delivery. Episodes with onset in pregnancy or later in the postpartum did not show a relationship with parity. These findings are in agreement with previous research (Forty et al., 2006) that provided evidence for an onset specifier for research limited to the first 8 weeks following delivery. In clinical practice there may be good reasons for extending the definition of postpartum episode to 6 months or beyond, but these results provide further evidence suggesting that research aimed at uncovering the aetiology of postpartum triggering should be focused on episodes with early onset.

#### 4.3.1 Differences between bipolar I, bipolar II and recurrent major depression

We have demonstrated an influence of parity on postpartum mood episodes in women not only with BD-I but also RMD. In bipolar women, however, the effect is limited to episodes of manic/mixed polarity whereas in unipolar depression, in which by definition episodes of high mood have not occurred, there is an influence on episodes of depression with early onset following delivery. An interesting question for further work is whether these early onset depressive episodes in RMD, while not meeting criteria for a mixed episode or hypomania, show evidence of bipolar features such as irritability, agitation and poor response to antidepressants. It is increasingly recognized that there is a spectrum of bipolarity and that some with depression while not meeting formal criteria for a mixed episode may show some bipolar symptoms. Indeed we know from our previous work that episodes of depression with onset within 4 weeks of delivery are a marker of underlying bipolarity with a higher risk of subsequent conversion to a bipolar diagnosis (Munk-Olsen et al., 2012).

It is also of note that there appeared to be no relationship between parity and perinatal episodes of any type in women with BD-II. In this regard, BD-II appears to have a different relationship to the childbirth trigger than BD-I and RMD.

### 4.4 Implications for clinical practice

For women with mood disorders, particularly for those with bipolar disorder, difficult decisions need to be made in relation to pregnancy and childbirth. Any information that can help to individualize the risk of illness may be useful. For women with BD-I, the odds of postpartum psychosis following a first pregnancy are double those for further deliveries. If a woman has remained well after their first baby, this provides some reassurance in subsequent pregnancies. However, it is important to remember that the risk of postpartum recurrence is very high (>60%) in women who have already experienced an episode of postpartum psychosis (Robertson et al., 2005).

### 4.5 Limitations

Our results need to be interpreted in the light of several limitations.

(1) Information about perinatal episodes was collected retrospectively. It should be noted, however, that the reporting of episodes at interview was in agreement with the medical records and the recollection of episodes of illness in relation to childbirth has been shown to be excellent (Cox et al., 1984).
(2) Pregnancies that occurred before the onset of mood disorder were included in the analyses. However, this represents a conservative bias, making it less likely that we would find an effect of primiparity as subsequent pregnancies are increasingly likely to have occurred following the onset of mood disorder.
(3) Recent evidence from clinical, epidemiological and molecular studies has challenged the current clear-cut unipolar–bipolar distinction. To improve the validity and reliability of the phenotypes, we excluded from the original study on RMD women who had ever experienced mood incongruent psychosis or psychosis outside of mood episodes. Although our approach reduced the variability and the risk of misdiagnosis, the low numbers of postpartum psychotic episodes in the RMD group may be due to sample characteristics and not be representative of the broad clinical spectrum of women presenting major depressive symptoms in clinical settings.
(4) Mothers who have had a previous episode of postpartum psychosis and want to extend their families may be aware of the risk of recurrence and take medication to prevent or promptly treat an emerging postpartum episode. We did not have detailed information on the drug management of the women and could not, therefore, establish the effect of medication in reducing the risk of a perinatal relapse. It is difficult to provide an accurate estimate of medication use in pregnant women with bipolar disorder. Because of the remitting and relapsing course of bipolar disorder, many women with bipolar disorder may not be taking medications or be in contact with psychiatric services (Jones and Craddock, 2005). Estimates from naturalistic prospective studies conducted in specialized centers vary. A study conducted in the US reported that only about 31% of women with bipolar I disorder maintained mood stabilizer treatment during pregnancy (Viguera et al., 2007), while studies from Europe have found higher rates of medication being used in pregnancy in bipolar women (75% in Bergink et al., 2012, 63% in our prospective study on bipolar disorder in pregnancy, data unpublished). However, the evidence suggests the increased risk of recurrence following delivery does not appear to be merely a result of stopping mood-stabilising medication (Viguera et al., 2000).

## 5 Conclusions

The results of our study indicate that in women with BD-I, episodes of postpartum psychosis are associated with first pregnancies. Episodes of depression in the postpartum are associated with primiparity only in mothers suffering from RMD and we did not find any effect of parity on perinatal episodes in mothers with BD-II. The primiparity effect was not due solely to women with postpartum episodes not going on to have further children.

Both biological and psychosocial factors may underpin the link with parity. First pregnancies may be a greater psychosocial stressor but there are also significant biological differences that may also play a role. These are important candidates for further study.

## Role of funding source

The sponsors (NISCHR, Wellcome Trust, the Stanley Medical Research Institute and the Medical Research Council) had no role in study design; in the collection, analysis and interpretation of data; in the writing of the report; or in the decision to submit the paper for publication.

## Conflict of interest

The authors report no conflicts of interest.

## Figures and Tables

**Table 1:** Participants characteristics by lifetime diagnosis.

**Lifetime diagnostic group**	**Bipolar I disorder**		**Bipolar II disorder**		**Recurrent major depression**	
	**(*****N*****=934)**		**(*****N*****=278)**		**(*****N*****=455)**	
	**Mean**	**SD**	**Mean**	**SD**	**Mean**	**SD**
Age at the interview	47.8	11.34	47.6	11.89	48.3	11.78
Age at onset	23.6	8.15	21.6^a^	8.45	24.4	7.92
Age at first pregnancy	25	5.01	25.3	5.55	24.4	5.2

	Median	Range	Median	Range	Median	Range
Deliveries	2^b^	1–7	2	1–6	2	1-8

**Pregnancies analysed**	***N*****=1761**		***N*****=568**		***N*****=1016**	
	***N***	**%**	***N***	**%**	***N***	**%**

Perinatal periods affected by mania/psychotic depression	480	27.2	24	4.3	20	2.0
Perinatal periods affected by non-psychotic depression	423	24.0	226	39.8	416	40.9

a Tukey multiple comparisons of means: BD-I vs. BD-II −2.06 (95% CI −3.39 to −0.73), adjusted *p*<0.001; RMD v BD-II 2.85 (95% CI 1.38 to 4.33), adjusted *p*<0.001.

b Wilcoxon rank sum test with continuity correction: BD-I vs. BD-II W=101,640.5, *p*<0.001; BD-I vs. RMD W=156736, *p*<0.001.

**Table 2:** Summary of the associations between parity and mood disorders in parous women with at least 2 live birth deliveries.

Lifetime diagnosis	Perinatal diagnosis	Onset in pregnancy	Onset within 6 weeks postpartum	Onset between 6 weeks and 6 months postpartum
BD-I	Mania or psychosis	–	OR 2.0	–
			95%CI 1.64–2.56	
			*p*=0.001	
BD-II	Major depression	NS	NS	NS
RMD	Major depression	NS	OR 1.8	NS
			95%CI 1.49–2.25	
			*p*=0.003	

Abbreviations and symbols: NS: not significant, OR: odds ratio; – analysis not conducted due to the small sample size.
